# Delayed ipsilateral intestinal perforation after endoscopic gastrojejunostomy: A perspective on underlying mechanisms

**DOI:** 10.1002/deo2.229

**Published:** 2023-04-05

**Authors:** Juan Reyes Genere, Zoilo K. Suarez, Vladimir Kushnir

**Affiliations:** ^1^ Department of Medicine Washington University School of Medicine Saint Louis USA; ^2^ Department of Internal Medicine Florida Atlantic University Charles E. Schmidt College of Medicine Boca Raton USA

**Keywords:** endoscopic ultrasonography, gastrojejunostomy, gastric outlet obstruction, intestinal perforation, pancreas cancer

## Abstract

The endoscopic management for malignant gastric outlet obstruction has expanded with the advent of endoscopic ultrasound‐guided gastrojejunostomy. Delayed perforation after endoscopic ultrasound‐guided gastrojejunostomy is an important yet rarely reported adverse event, and it is unclear why some patients may suffer this complication. We present a case of intestinal perforation occurring one month after endoscopic ultrasound‐guided gastrojejunostomy and discuss potential mechanisms which may contribute to this outcome.

## INTRODUCTION

Malignant gastric outlet obstruction (GOO) is an important manifestation of advanced foregut cancer, associated with deterioration in the quality of life and reduced survival. Enteral stenting (ES) and surgical gastrojejunostomy are effective methods for relieving GOO and restoring the flow of gastric chyme into the small bowel. However, issues with therapeutic durability and confinement of the biliary outflow with ES, as well as peri‐operative risk for surgery, represent limitations to these established options.^1^, ^2^

Endoscopic ultrasound‐guided gastrojejunostomy (EUS‐GJ) is a technique gaining popularity as an alternative therapy for GOO. EUS‐GJ has the advantage of creating a gastrojejunal conduit that bypasses the area of disease, resulting in a more durable treatment that does not interfere with the biliary system. EUS‐GJ has now matured into a phase of technical standardization and holds promise in becoming the preferred treatment for GOO.^3^ Yet, EUS‐GJ is a newer procedure; and while immediate safety concerns have been addressed, data on delayed safety is limited to a few cohorts.

## CASE REPORT

**FIGURE 1:**
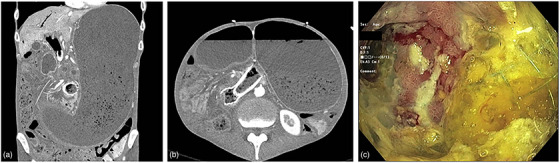
Computed tomography scan demonstrating severe gastric distention and prior biliary and enteric stents in situ (a and b). Endoscopic image of the enteric stents with tissue ingrowth (c).

**FIGURE 2:**
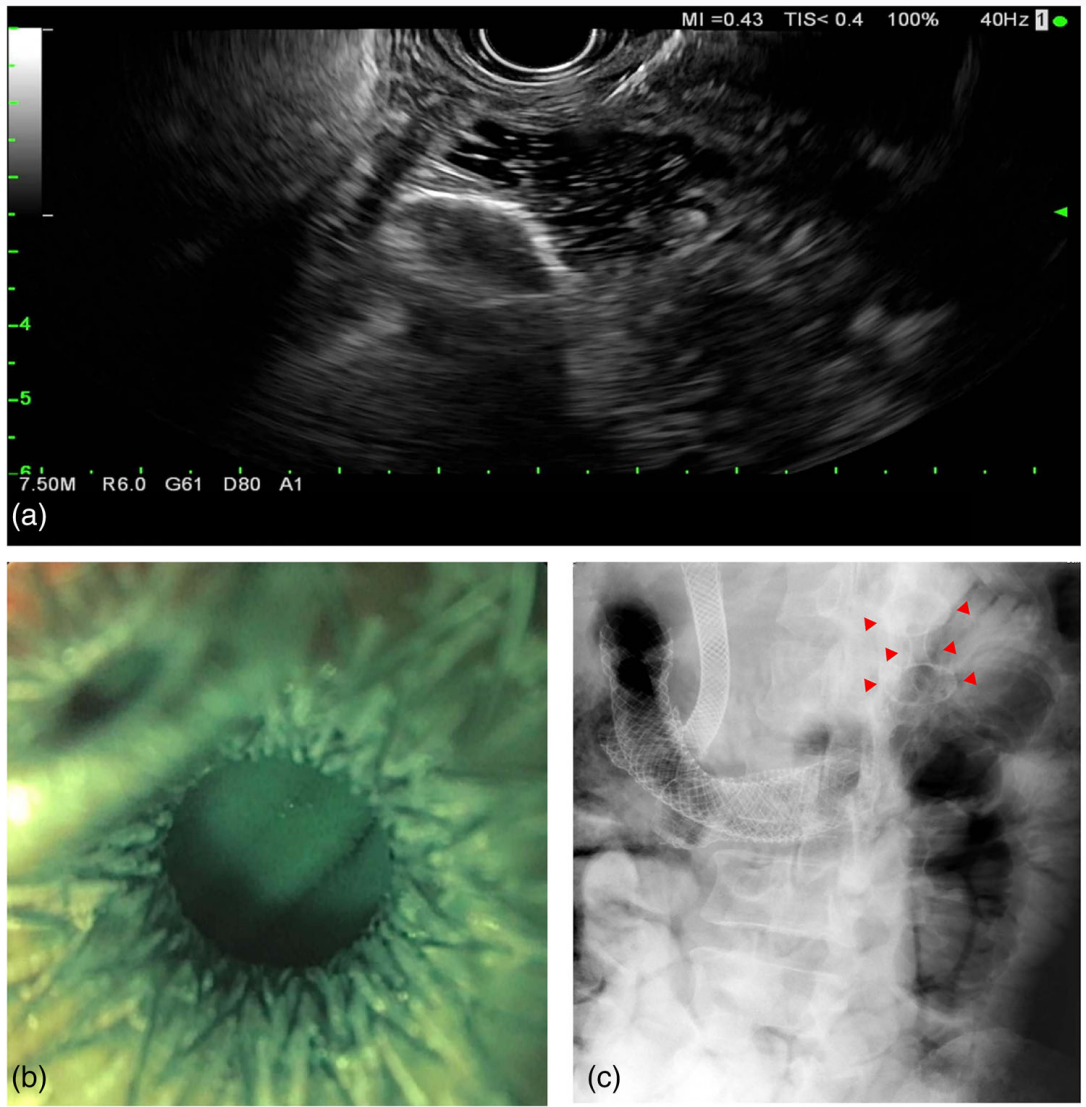
Endoscopic ultrasound‐guided gastrojejunostomy procedure using nasojejunal tube‐assisted technique and an electrocautery‐enhanced lumen apposing metal stent. Stent placement under real‐time endosonographic imaging (a). Endoscopic visualization of the small bowel after successful stent placement with drainage of blue‐tinted sterile saline (b). Radiographic image confirming positioning of the lumen apposing stent distal to the enteric stents ‐ indicated by red arrows (c).

A 48‐year‐old male with unresectable pancreatic adenocarcinoma complicated by biliary obstruction managed by stenting and GOO with prior ES presented with recurrent GOO. Nine weeks prior to presentation, the patient underwent initial ES placement to treat a malignant stenosis located in the second and third portions of the duodenum. He had done well until two weeks prior to the presentation, when he experienced recurrent GOO due to aboral ES migration. An overlapping co‐axial ES was placed, however, the patient developed recurrent GOO one week prior to presentation due to another aboral migration of the internal, overlapping stent. At this time, a third co‐axial ES was placed with the proximal flange positioned in the duodenal bulb. Despite these efforts, he continued to have GOO and it was felt he had developed concurrent progression of gastric contractile dysfunction secondary to late‐stage malignant GOO (Figure 1). The patient was referred for salvage EUS‐GJ with the intention to create a shorter conduit to facilitate the passage of gastric chyme. Following 24‐hr of nasogastric tube decompression, EUS‐GJ was accomplished via the nasojejunal tube‐assisted technique (Figure 2). This method utilizes an 8.5 Fr nasobiliary tube, which is passed into the jejunum fluoroscopically over a guidewire. The tube is attached to a foot‐pedal irrigation pump to infuse 500–800 cc of sterile saline, contrast, and methylene blue mixture into the small bowel. Concurrently, an intravenous dose of 0.5 mg glucagon is administered to temporally suppress peristalsis and facilitate adequate small bowel visualization. Next, a linear echoendoscope is advanced into the stomach. An avascular location for stent insertion was chosen that was distant to the primary tumor, did not have intervening ascites, and measured 7.4 mm between the jejunal and gastric mucosal surface. A 15 mm electrocautery‐enhanced lumen apposing stent (Hot AXIOS; Boston Scientific Corporation, Marlborough, MA, USA) was used for EUS‐GJ creation and the tract was dilated with an 8 mm balloon. Following successful EUS‐GJ, the patient's GOO was relieved and he began tolerating a full‐liquid diet the next day.

**FIGURE 3:**
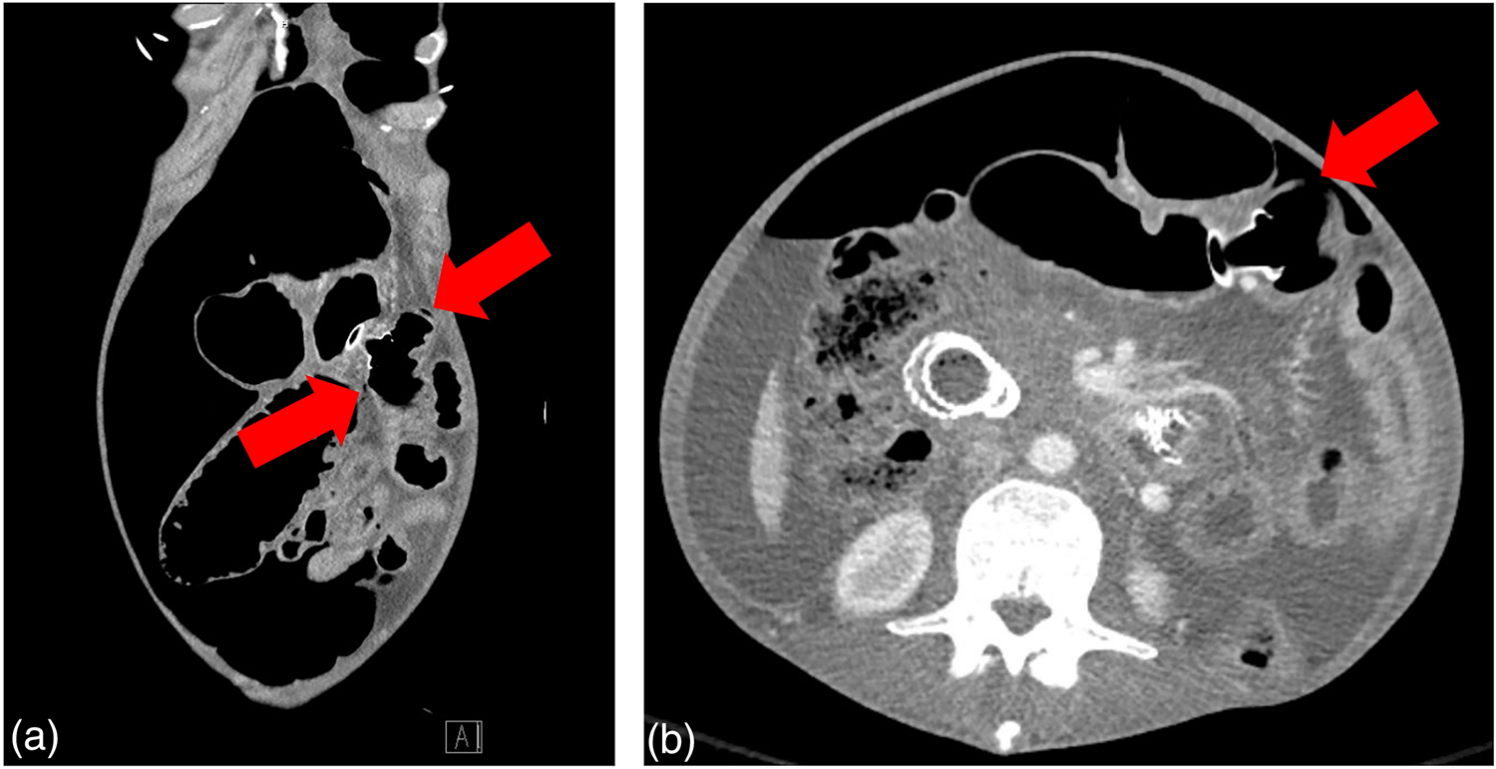
Computed tomography scan with flanking jejunal ulcerations indicated by arrows (a). The ulceration indicated on the patient's left side was perforated and this is better demonstrated on the cross‐sectional image indicated by an arrow (b).

He was started on a standard full‐dose daily proton pump inhibitor and later dismissed from the hospital. At a post‐admission clinic visit, he graduated to a soft diet two weeks after EUS‐GJ and was gaining weight. Four weeks after EUS‐GJ, he presented to the emergency department with acute abdominal pain and distention. A computed tomography scan revealed pneumoperitoneum and a jejunal defect adjacent to the lumen‐apposing metal stent (LAMS) (Figure 3). Exploratory laparotomy was notable for two flanking jejunal ulcerations, 1 cm lateral to the adjacent stent flange, one of which was confirmed to be perforated. Peritoneal carcinomatosis was appreciated without direct involvement of the perforated section of the bowel. The LAMS was removed, and the jejunal defect was closed. A surgical gastrostomy tube was placed for palliative decompression, and the patient expired after instituting comfort measures.

## DISCUSSION

EUS‐GJ for malignant GOO has many advantages over conventional methods. A primary barrier to the widespread adoption of EUS‐GJ may be largely due to the perceived risk for perforation. Therefore, substantial efforts have focused on addressing these concerns through the development of a standardized technique, identifying factors influencing technical success, and characterization of safety events and their potential for endoscopic remediation.^3^, ^4^, ^5^

Delayed intestinal perforation after EUS‐GJ appears to be rare and mostly reported in single‐case reports (Table 1). When delayed perforations occur in this setting, they have been described to occur in the contralateral jejunal wall.^6^, ^7^ McKinley and colleagues propose a sensible explanation. Chronic LAMS‐mediated pressure ischemia onto the opposing jejunum leads to ulceration and subsequent perforation.^6^ In our case, however, the jejunal perforation occurred on the ipsilateral wall of the LAMS. The operative findings also confirmed that there were two jejunal ulcerations, and these were located 1 cm from the outer flange of the LAMS on the ipsilateral jejunum. These findings suggest a mechanism for mucosal injury that is distinct from the aforementioned reports. Specifically, the pattern of injury that we observed is more consistent with recurring and transient, jejunal‐stent invagination pressure injury.

**TABLE 1:** Summary of studies reporting delayed perforation after endoscopic gastrojejunostomy

**Study author (s)**	**Study dates**	**Country**	**Type of study**	***N***	**Perforation totals**	**Early perforation** **^†^**	**Delayed perforation** **^‡^**
Abbas et al^3^	2016–2020	USA	Single‐center prospective cohort study	50 EUS‐GJ	2 (4%)	1	1
McKinley et al.^6^	2021	USA	Case report	1	1 (N/A)	0	1
Taibi et al.^7^	2019	France	Case Report	1	1 (N/A	0	1
Perez‐Cuadrado‐Robles et al.^10^	2020– 2022	France	Retrospective case‐control study	28 EUS‐GJ included in the final analysis	2 (7.1%)	1	1

^†^ Early perforation defined as <14 days from procedure.

^‡^ Delayed perforation defined as ≥14 from index procedure.

Since most patients do not suffer delayed perforation after EUS‐GJ, it is likely that this is a manifestation of a multifactorial process that begins with ulcerogenesis. Several facultative (i.e., tobacco use, drugs, and stress) and other risk and protective factors are known to impact the integrity of the gastrointestinal mucosa. Stent implantation introduces an additional risk factor that is felt to be related to chronic stent‐related pressure ischemia, and this may manifest in different patterns of injury. Perforation is a severe complication of gastrointestinal ulcers, and studies have implicated smoking tobacco, nonsteroidal anti‐inflammatory drugs (NSAID), older age, and alcohol use with perforated peptic ulcer disease.^8^ Our patient was an active smoker, and this could have contributed to his risk, but he did not exhibit other risk factors for peptic ulcer disease or perforation. Furthermore, he was receiving full dose proton pump inhibitor therapy. The impact of progressive carcinomatosis is curious, given that bowel fixation may intuitively increase the chance of stent contact injury. While limited data exist for this patient population, no delayed EUS‐GJ perforations have been reported in carcinomatosis, including from a recently published study.^9^ From the operative standpoint, it's noteworthy to mention the distance between the stomach and small bowel was <10 mm and there was no evidence of intervening ascites – both factors associated with EUS‐GJ technical success.^5^ Additionally, delayed perforation is not associated with stent size, as prior reports have occurred with both 15 and 20‐mm diameter LAMS.^3^, ^6^, ^7^, ^10^ While factors of mucosal barrier integrity should be acknowledged, they do not entirely explain the prevalence of perforated peptic ulcer disease or, accordingly, perforation after stent implantation within the gastrointestinal tract. Other factors pertinent to long‐term safety may be unrecognized due to the low incidence of delayed EUS‐GJ perforations. Consequently, definitive guidelines have not been established to avoid this rare adverse event. We can expect that many years of additional collective experience will be required before a robust analysis of risk factors for delayed perforation after EUS‐GJ can be elucidated. Until then, optimizing the risk‐ and protective‐factor balance for ulcerogenesis is advised.

In conclusion, delayed perforation after EUS‐GJ is rare and is likely to be the result of multifactorial mechanisms. Chronic stent‐related pressure ischemia may play a central role in this process and manifest as direct contralateral or ipsilateral wall contact injury. While these mechanisms are not readily addressable, apart from stent re‐design, we should expect to learn more about EUS‐GJ safety over time. In the meantime, modifiable factors such as gastric acid suppression and mitigating tobacco, illicit drug, or NSAID use are actionable interventions that may minimize these events.

## CONFLICT OF INTEREST STATEMENT

None

## ETHICS STATEMENT

Institutional review board review is not required for case reports with no identifiable health information. Consent for publication of the submitted manuscript and associated images was obtained from the patient's next of kin. The patient was deceased at the time of manuscript preparation.
